# Evaluating new technology for biodiversity monitoring: Are drone surveys biased?

**DOI:** 10.1002/ece3.7518

**Published:** 2021-05-01

**Authors:** Evangeline Corcoran, Simon Denman, Grant Hamilton

**Affiliations:** ^1^ School of Biological and Environmental Sciences Queensland University of Technology Brisbane Qld Australia; ^2^ School of Electrical Engineering and Robotics Queensland University of Technology Brisbane Qld Australia

**Keywords:** artificial intelligence, automated wildlife detection, drones, machine learning, survey design, UAV, unmanned aerial vehicle, wildlife abundance

## Abstract

Drones and machine learning‐based automated detection methods are being used by ecologists to conduct wildlife surveys with increasing frequency. When traditional survey methods have been evaluated, a range of factors have been found to influence detection probabilities, including individual differences among conspecific animals, which can thus introduce biases into survey counts. There has been no such evaluation of drone‐based surveys using automated detection in a natural setting. This is important to establish since any biases in counts made using these methods will need to be accounted for, to provide accurate data and improve decision‐making for threatened species. In this study, a rare opportunity to survey a ground‐truthed, individually marked population of 48 koalas in their natural habitat allowed for direct comparison of the factors impacting detection probability in both ground observation and drone surveys with manual and automated detection. We found that sex and host tree preferences impacted detection in ground surveys and in manual analysis of drone imagery with female koalas likely to be under‐represented, and koalas higher in taller trees detected less frequently when present. Tree species composition of a forest stand also impacted on detections. In contrast, none of these factors impacted on automated detection. This suggests that the combination of drone‐captured imagery and machine learning does not suffer from the same biases that affect conventional ground surveys. This provides further evidence that drones and machine learning are promising tools for gathering reliable detection data to better inform the management of threatened populations.

## Graphical abstract

Wildlife surveys using drones are becoming increasingly popular, but little is known about the factors influencing detection of threatened species. We show a range of factors can influence the detections of koalas in a natural setting using traditional survey methods and drone surveys using manual evaluation of the imagery. Drone surveys combined with AI algorithms were not affected by these factors, suggesting that they provide a less biased approach to threatened species detection.

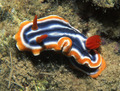

## 1 INTRODUCTION

The detection of individual animals remains the cornerstone of wildlife monitoring and abundance estimation, particularly for threatened species. For terrestrial species, surveys have typically been carried out by researchers on the ground. Surveys are often organized as line transects or point counts and rely on the visual detection of target species (Dique et al., 2003; Scheele et al., 2018). The advent of new technologies such as drones, in combination with appropriate sensors and machine learning algorithms for automated detection, has the potential to revolutionize wildlife surveys (Hollings et al., 2018). Drones can cover large areas more rapidly than ground surveyors, often where it may not be feasible or practical for ground‐based researchers to conduct surveys (Koh & Wich, 2012; Seymour et al., 2017). While the data captured by drones have most often been analyzed manually, machine learning‐based methods are being used with increasing frequency to automated detection (Corcoran et al., 2019; Lhoest et al., 2015; Longmore et al., 2017; Oishi et al., 2018; Seymour et al., 2017). This has led to faster and more accurate counts of wildlife population with fewer false‐negative results compared to manual image analysis and conventional ground surveys (Corcoran et al., 2019; Gonzalez et al., 2016; Lhoest et al., 2015; Longmore et al., 2017; Oishi et al., 2018; Seymour et al., 2017).

Given the surge in the use of drones, machine learning, and their potential importance in conservation programs, it is important to establish their limits and any possible biases. For example, in traditional surveys, accounting for the probability of detection of individual animals is fundamental for accurate abundance estimation (Royle & Dorazio, 2006). Unaccounted for biases in detection of species introduced by new survey methods could mask declines if the new methods result in an increased probability of detection compared to currently used methods (Kery & Schmidt, 2008). Conversely, they could erroneously suggest declines if they result in decreased probability of detection compared to currently used methods (Kery & Schmidt, 2008). Differences among individuals due to demographic traits, behaviors, and habitat selection can lead to hidden biases that result in inaccurate counts of populations when using traditional survey methods (Biro, 2013; Carter et al., 2012; Merrick & Koprowski, 2017). For example, the abundance of wolves in France was found to be underestimated by 27% when individual differences in detectability were not considered (Marescot et al., 2011).

While efforts have been made to understand variables that influence detection probabilities in drone surveys (Baxter & Hamilton, 2018), as yet there have been no studies that have been able to robustly compare these factors with traditional survey methods. Traits such as sex, age, and reproductive status and behaviors such as movement, burrowing, and microhabitat preferences that differ among individual animals have been shown to impact on detection probability in remotely sensed camera trap data (Karp, 2020; Meek et al., 2014, 2016). These factors are important to consider, as they may lead to certain demographics of a population being less likely to be detected and therefore under‐represented in counts. This leads to underestimations of abundance, with important conservation implications (Brack et al., 2018; Karp, 2020; Meek et al., 2014, 2016).

We explore this issue by comparing the factors that impact on the probability of detection of koalas (*Phascolarctos cinereus*) observed by experts from the ground to the factors that impact on the probability of detection for the same koala population data using manual and automated analysis of thermal images collected from drones. Koalas are a vulnerable Australian mammal species that are cryptic, widespread, and often occur in inaccessible areas (Beyer et al., 2018; McAlpine et al., 2015). Automated analysis of drone‐derived thermal imagery of koalas has been shown to be more accurate than ground‐based detections (Corcoran et al., 2019) and to be useful for koala detection across a range of environments (Hamilton et al., 2020). The impact of individual traits and behaviors such as sex and host tree preferences on detection of koalas using this method and how these detectability variables compare to those impacting on ground observation and manual analysis remain unknown (Corcoran et al., 2019; Ellis & Bercovitch, 2011). Gaining an understanding of this will provide insight into how individual differences may bias automated detection of wildlife in drone imagery more broadly and better inform the use of these new technologies so that reliable data on vulnerable populations can be gathered from which to make effective management decisions.

## 2 METHODS

More detailed description of the survey design, manual, and automated image analysis methods used collect data for model development can be found in Corcoran et al. (2019). In summary, the dataset for this study consisted of 119 observations of individually identified radio‐collared koalas surveyed at two sites in Petrie, Queensland. The north site was approximately 1.1 km by 0.5 km in size, and the south site was approximately 1.0 km by 0.7 km. The radio‐collared koalas were surveyed 5 times between May and August 2018 using a FLIR Tau 2 640 thermal infrared sensor (FLIR) mounted to the underside of a Matrice 600 Pro drone and A3 flight controller, allowing an overhead view of the canopy. The drone flew at a fixed speed of 8 m per second and at a fixed height of 60 m above the ground (approximately 30 m above the top of the canopy) in a preprogramed pattern of transects 20 m apart, covering the entirety of the survey sites. All surveys were conducted during predawn hours in temperatures ranging from 8.0–21.7°C to ensure maximum contrast between the body temperature of koalas and their surroundings (Corcoran et al., 2019). On the same day that each of the five drone surveys was conducted, ground surveys were conducted by expert observers, resulting in a total of five ground surveys. All radio‐collared koalas present within the study sites at the time of surveying were tracked using conventional radio telemetry and their GPS locations recorded. Due to koalas being free to move in and out of the boundaries of the study sites, this meant that some individual koalas were observed in all or multiple surveys and some in only one survey.

Generally, determining the factors impacting on probability of detection can be challenging because of the difficulty in disentangling availability (whether the individual animal is present in the area and so available to be detected or not) with the capacity to detect the individual once it is available (Brack et al., 2018). Because the koalas in this study are radio‐collared, their exact location is available. This means we can know with certainty that a nondetection occurs because they are present but unable to be seen. Ground observers thus assigned a visibility score that can be considered an independent proxy for ground‐based detection probability. This visibility score was assigned by ground observers to each individual koala identified within the site during each survey given on a scale of 0‒5, with 0 being completely obscured from view of the ground observer at all angles, and five being completely unobscured from all angles. No visibility scores of 5 were allocated by the ground observers at any time, reducing this to a 5‐point scale from 0 to 4.

The identity, sex, and height in tree of individual koalas were recorded as well as the genus, diameter at breast height (DBH), and height of host trees. The height of host trees and height of koalas in host trees was measured using a clinometer. The height of koalas from the top of host trees was then calculated by subtracting the height of koalas from the total height of host trees. Tree genus was divided into categories of *Corymbia* spp., *Eucalyptus* spp., and “Other” which included tree species of the *Casuarina*, *Lophostemon*, *Ulmus*, *Melaleuca*, *Acacia*, *Cinnamomum,* and *Alphitonia* genera.

The drone‐derived thermal images were analyzed both manually, by a researcher evaluating the footage frame by frame and recording instances and co‐ordinates of possible koalas, and using the automated koala detection algorithm described full in Corcoran et al. (2019). This algorithm pipeline involved a combination of two previously published deep convolution neural networks referred to as Faster R‐CNN and YOLO, the results of which were fused so that only objects that were detection above a set threshold of certainty (0.05) were recorded as possible koalas (Corcoran et al., 2019; Redmon & Farhadi, 2016; Ren et al., 2015). The authenticity of possible koalas identified in both manual and automated image analysis was verified by comparing their co‐ordinates to those of the radio‐collared koalas confirmed to be on site by the telemetry‐guided ground observers. It was then recorded whether the radio‐collared koalas found to be present within the survey site at the time of ground and drone surveying were successfully detected in manual or automated image analysis (1) or not detected (0).

Using Shapiro–Wilk tests for normality, the height of host trees (*W* = 0.98, *p* = .14) and height of koalas above ground (*W* = 0.98, *p* = .08) were found to be normally distributed, while the height below the top of the host tree (*W* = 0.90, *p* = 1.84^e−06^) and DBH of host trees (*W* = 0.82, *p* = 2.56^e−09^) were not normally distributed. Ordinal regression models were therefore developed to investigate the impact of covariates on detectability of individual radio‐collared koalas to ground observers, as the response variable was ordered categorical data, the five‐point scale visibility rating and the some of the covariates investigate were not normally distributed. These models were constructed using the “MASS” package “polr” function in R (Venables & Ripley, 2002). Because the response variable for manual and automated analysis of drone images was a binary outcome (detected = 1, undetected = 0) and some of the covariates investigated were not normally distributed, generalized linear models (GLMs) with binomial error distributions and logit link functions were developed for probability of detection for these methods using the “lme4” R package (Bates et al., 2015).

Separate models for detectability of koalas by ground researchers, in manual, and in automated analysis of thermal images derived from the drone were developed using the same training dataset, which was comprised of a random 80% split of the total dataset (*n* = 95). The data were split into separate training and testing datasets in this way to ensure predictions made with the final models were not biased by predicting values for data they had already been trained on. Models of probability of detecting koalas with each method were developed separately in a forward stepwise manner. First, univariate models were evaluated for each variable listed in Table 1 and ranked by Akaike information criterion (AIC), residual deviance, and *p*‐value. Interaction variables between host tree height and koala height above ground, host tree height and koala height below treetop, and host tree height and host tree diameter at breast height were also investigated as possible covariates. Subsequently, multivariate models with all possible combinations of covariates found to have a significant *p*‐value in univariate model testing were evaluated. A covariate was only retained in the final model if it resulted in a reduction of AIC of greater than or equal to two compared to the model with the next best fit (Bozdogan, 1987; Terletzky & Koons, 2016). McFadden's pseudo *R*‐square values were then calculated for the final models in order to determine the percentage of variation in the response variable that could be explained by the selected model covariates.

**TABLE 1:** List of covariates used in models of probability of manual and automatic detection of koalas in RPAS‐derived thermal imagery, and visibility rating given to koalas by ground observers

Covariate	Unit of measurement
Sex	Categorical (Male, Female)
Height of koala above ground	Meters (m)
Distance of koala from top of host tree	Meters (m)
Host tree genus	Categorical (*Corymbia*, *Eucalyptus*, Other)
Host tree diameter at breast height	Centimeters (cm)
Host tree height	Meters (m)
Observer (ground observation only)	Individual identification code of ground observer

The final models were then used to predict the probability of detection for koalas in manual and automated image analysis and which visibility rating they would receive from ground observers. The classification rate of each was calculated as the proportion of test data correctly classified as either “undetected” or “detected” (manual and automated analysis of drone‐derived thermal images) or into the correct visibility score from 0–4. The dataset used to evaluate predictions made based on the final models was comprised of the remaining 20% of randomly split data (*n* = 24).

To examine the differences in host trees used by males and females, *t* tests were conducted to compare the difference in mean host tree height and height above ground between sexes, while Mann–Whitney *U* tests were conducted to compare the difference in mean DBH of host trees and height of koalas below tree tops.

## 3 RESULTS

The training dataset was comprised of observations of 59 female and 36 male koalas. There was no significant difference in the mean height of host trees male, and female koalas were found in during surveys (*t* = 0.07, *df* = 63.87, *p* = .95). There was no difference found between the mean height above ground at which male (13.53(±5.28)) and female (14.80(±4.44)) koalas were positioned in host trees during surveys (*t* = 1.20, *df* = 64.40, *p* = .23) or the mean height of male (4.86(±3.97)) and female (3.68(±2.92)) koalas from the top of host trees (*W* = 881, *p* = .16). There was no difference in mean diameter at breast height of host trees used by male 62.64(±45.36) and female 49.15(±24.99) koalas during surveys (*W* = 928, *p* = .31). Male and female koalas were not significantly associated with any host tree genus (Chi‐squared = 4.35, *df* = 2, *p* = .11).

The sex of koalas was found to significantly impact the visibility rating assigned to individuals by experienced ground observers (*p* < .01). Male koalas were more likely to receive higher ratings of 3 or 4 compared to females, which were more likely to receive lower ratings of 1 or 2. Overall, male koalas were found to be 3.817 (*β*_koala sex_ = 1.16 ± 0.41) times more likely to receive a higher visibility rating than females.

Tree genus was also found to significantly affect the visibility of koalas to ground observers, specifically when comparing *Corymbia* spp. host trees to *Eucalyptus* spp. host trees (*p* = .02). Visibility of koalas was similar in both *Eucalyptus* spp. and trees of “other” genera, with koalas in *Eucalyptus* trees 3.50 (*β*_Eucalyptus_ = 1.25 ± 0.52) and 3.31 (*β*_Other_ = 1.20 ± 0.62) more likely to receive a higher rating than koalas in *Corymbia* spp.

Height of koalas above ground was also found to have a significant impact on the visibility rating given to individuals by ground observers (*p* = 1.35^e−05^). The visibility rating given to koalas was more likely to be lower when koalas were positioned higher above the ground with the likelihood that the visibility score would increase by 1 increasing 0.82 times for every meter increase in height (*β*_koala height above ground_ = −0.20 ± 0.05). This meant that koalas that were positioned higher above the ground during surveys were more difficult for ground‐based observers to detect.

Finally, host tree height significantly impacted on the visibility rating given to koalas by ground observers (*p* < .01). For every meter increase in host tree height, it was 0.903 times as likely that the visibility score would increase by 1 (*β*_Host tree height_ = −0.10(±0.04)) resulting in koalas receiving lower ratings in taller trees. Host tree diameter at breast height (*p* = .36), distance of koalas from the top of host trees (*p* = .23), and the identity of the observer who assigned the score (*p* = .28) were all not found to be significant in determining the visibility rating ground observers gave koalas.

The top‐performing model of visibility rating for koalas observed by ground observers was a multivariate ordinal regression model with koala height above ground, koala sex, and host tree genus as covariates (Table 2). This model explained 14.95% of variation in visibility score (McFadden's pseudo *R*‐squared = 0.15) and achieved a classification rate of 54.17% for the test dataset.

**TABLE 2:** Models of visibility score assigned to koalas by ground observer ranked by AIC

Covariates	Null deviance	Residual deviance	AIC
Koala height above ground + Koala sex + Host tree genus	232.52	197.77	211.77
Koala height above ground + Koala sex	232.52	204.12	214.12
Koala height above ground + Host tree genus	232.52	202.91	214.91
Koala height above ground	232.52	211.64	219.64
Koala height above ground + Host tree height	232.52	210.30	220.30
Host tree height	232.52	223.52	231.52
Koala sex	232.52	224.12	232.12
Host tree genus	232.52	226.13	236.13
Interaction between host tree height and distance of koala from treetop	232.52	230.29	238.29
Null model	232.52	232.52	238.52
Interaction between host tree height and host tree DBH	232.52	230.64	238.64
Distance of koala from treetop	232.52	231.07	239.07
Host tree DBH	232.52	239.69	239.69
Interaction between host tree height and koala height above ground	232.52	232.49	240.49
Observer	232.52	226.68	240.68

Tree genus significantly impacted the probability of manually detecting koalas using thermal imagery, specifically when comparing trees of “*other*” genera to *Corymbia spp*. trees (*p* < .01). Koalas in *other* trees were 0.11 times as likely to be detected than koalas in *Corymbia* trees (*β*_Other_ = −2.23 ± 0.79). Host tree height also significantly impacted on probability of manual detection (*p* = .04). For every meter increase in host tree height, koalas were 1.08 times as likely to be detected (*β*_Host tree height_ = 0.081(±0.039)); therefore, koalas resting in taller trees during drone surveys were more likely to be detected using manual analysis than koalas in shorter host trees. Koala sex (*p* = .48), host tree DBH (*p* = .79), height of koalas from ground (*p* = .12), and distance of koalas from tree tops (*p* = .18) had no significant effect on probability of manual detection of koalas in RPAS‐derived thermal imaging.

The top‐performing model of probability of manual detection was a univariate ordinal regression with host tree genus as the explanatory variable, as the multivariate model with host tree genus and tree height did not provide a lower AIC (Table 3). This model explained 8.0% of variation in visibility score (McFadden's pseudo *R*‐squared = 0.08) and achieved a classification rate of 41.67% for the test dataset.

**TABLE 3:** Models of probability of manually detecting koalas in drone‐derived thermal images ranked by AIC

Covariates	Null deviance	Residual deviance	AIC
Host tree genus	126.07	115.99	121.99
Host tree genus + Host tree height	126.07	114.48	122.48
Host tree height	126.07	121.54	125.54
Interaction between host tree height and koala height above ground	126.07	122.79	126.79
Koala height above ground	126.07	123.58	127.58
Null model	126.07	126.07	128.07
Distance of koala from treetop	126.07	124.16	128.16
Host tree DBH	126.07	124.87	128.87
Interaction between host tree height and host tree DBH	126.07	125.30	129.30
Koala sex	126.07	125.56	129.56
Interaction between host tree height and distance of koala from treetop	126.07	125.92	129.92

In contrast to ground‐based observations and manual detections using drone‐captured thermal imagery, none of the covariates investigated explained a significant amount of the variation in probability of detecting koalas in thermal images derived from drones using automated methods (Table 4).

**TABLE 4:** Models of probability of automatically detecting koalas in drone‐derived thermal images ranked by AIC

Covariates	Null deviance	Residual deviance	AIC	*p* value
Null model	59.54	59.54	61.54	N/A
Host tree height	59.54	58.31	62.31	.28
Koala height above ground	59.54	58.34	62.34	.28
Interaction between host tree height and host tree DBH	59.54	59.23	63.23	.59
Koala sex	59.54	59.36	63.36	.67
Interaction between host tree height and distance of koala from treetop	59.54	59.37	63.38	.68
Host tree DBH	59.54	59.39	63.39	.69
Distance of koala from treetop	59.54	59.43	63.43	.74
Host tree genus	59.54	59.45	65.45	.77 (*Corymbia* | *Eucalyptus*),. 91 (*Corymbia* | other)
Interaction between host tree height and koala height above ground	59.54	59.49	63.49	.84

A summary comparison of the effects of each covariate on probability of detecting koala using ground observation, manual, and automated analysis of drone‐acquired thermal imagery can be found in Table 5.

**TABLE 5:** Comparison of effects and significance for each possible covariate in explaining variance in probability of detection koalas in traditional ground surveys, manual, and automated analysis of drone‐acquired thermal imagery

Covariate	Ground observation	Manual analysis of drone‐acquired thermal imagery	Automated analysis of drone‐acquired thermal imagery
Sex	**Females** significantly **less likely** to be detected than **males**	No significant effect on detection	No significant effect on detection
Height of koala above ground	Koalas positioned **higher above ground** significantly **less likely** to be detected than koalas **closer to the ground**	No significant effect on detection	No significant effect on detection
Distance of koala from top of host tree	No significant effect on detection	No significant effect on detection	No significant effect on detection
Host tree genus	Koalas in ***Corymbia* sp**. host trees significantly **less likely** to be detected than koalas in ***Eucalyptus* sp**. or **“other”** host trees	Koalas in **“other”** host trees significantly **less likely** to be detected than koalas in ***Eucalyptus sp***. and ***Corymbia* sp**. host trees	No significant effect on detection
Host tree diameter at breast height	No significant effect on detection	No significant effect on detection	No significant effect on detection
Host tree height	Koalas in **taller** host trees significantly **less likely** to be detected than koalas in **shorter** host trees	Koalas in **taller** host trees significantly **more likely** to be detected than koalas in **shorter** host trees	No significant effect on detection

## 4 DISCUSSION

A key finding of this study was that none of the traits of koalas or their host trees had a significant impact on the probability of detection of individuals in automated analysis of drone‐derived thermal imagery. Conversely, several of these factors significantly impacted on ground‐based detection by experts and even on manual detection of koalas in drone‐derived thermal imagery. This is significant in light of recent work that has proposed using drone thermal imagery for the detection of koalas in the absence of automated imagery (Beranek et al., 2020). The results of the current study suggest that attempts to detect koalas without machine learning approaches may yield significant biases. More broadly, this important finding suggests that drone‐based automated detection methods have the potential to overcome the biases in detection due to environmental and behavioral factors (Corcoran et al., 2019, 2020; Hamilton et al., 2020).

When using traditional ground‐based counts for koalas, this analysis suggests that female koalas are likely to be under‐represented. A possible explanation for this is the size difference between male and female koalas, as female koalas have been found to have a significantly lower average adult body mass of 6.2(±0.2) kg compared to 7.1(±0.3) kg for males, resulting in females making for smaller targets for ground observers to detect which are more easily obscured by foliage between the animal and the observer (Ellis & Bercovitch, 2011). It was also found that koalas higher in taller trees may have fewer detections, likely because they were further away from the ground observer. This may also explain why koalas in taller trees were more likely to be found in manual analysis of drone imagery as koalas higher above the ground were positioned closer to the overhead drone‐mounted camera. Tree species composition of a forest stand was found to impact on detection for both ground observation and manual image analysis methods.

A surprising result was that ground observers found it harder to detect koalas in *Corymbia* spp. host trees compared to trees of *Eucalyptus* spp. and other genera, given the morphological similarities between trees of the *Corymbia* and Eucalyptus genera (Vlasveld et al., 2018); further investigation revealed a higher ratio of female koalas in *Corymbia* host trees. This may explain a portion of the decreased detectability of animals in these trees as smaller female koalas were found to be more difficult to detect. However, as this covariate resulted in better model fit even when koala sex was already accounted for in the model, there are likely other attributes of *Corymbia* spp. host trees impacting on detection by ground observers that remain unaccounted for.

While drone data have the potential for false‐positive detections, methods for abundance estimation have been developed to account for this (Corcoran et al., 2020). A review by Kellner and Swihart (2014) found that despite numerous potential methods of accounting for imperfect detection in wildlife abundance and distribution estimates only 23% of studies (out of 537) accounted for these. This also reinforces the need for appropriate abundance modeling regardless of the technique used. Simultaneous collection of RGB as well as thermal images from drone surveys could also help reduce the number of spurious detections as after analysis of thermal imagery has been conducted to detect the heat signatures of cryptic animals, analysis of RGB images may provide more information on features of detected animals and their surroundings (McKellar et al., 2020). This additional information may then make it easier to identify duplicates and distinguish between species of similar size (Corcoran et al., 2021; McKellar et al.,^2020^). However, only manual analysis of simultaneously collected RGB and thermal imagery from drones has only been conducted thus far, and it remains unknown whether these two data streams could be integrated into automated image analysis methods due to difficulties synchronizing the images collected with RGB and thermal infrared sensors (Corcoran et al., 2021; McKellar et al., 2020).

The capture of thermal data from drones in combination with machine learning may have advantages over human observers due to the thresholds and features used by computer vision to identify koala heat signatures being different than the features used by the human eye, allowing for detection even in cases where animals are partially obscured (Lhoest et al., 2015; Longmore et al., 2017; Oishi et al., 2018; Seymour et al., 2017). This is an intriguing possibility considering that in this experiment, human observers were advantaged by knowing not only that koalas were available to be detected but because they tracked radio‐collared koalas; observers also had a reasonable idea of where to search in the canopy. Where radio telemetry is not used, we would expect ground‐based observers to perform worse than in this experiment. Compounding that, in traditional surveys observer expertise and fatigue can also reduce detections, where this has no impact on detection with machine learning (Lhoest et al., 2015; Longmore et al., 2017; Oishi et al., 2018; Seymour et al., 2017).

While this study has been conducted at a single site with several sampling events, and using a single species, the rapid increase in the number of drone surveys for threatened species using automated detection suggests that it is important to continue to comprehensively evaluate the methodology. All combinations of species and environment will have their own set of factors that will impact on detectability. However, as demonstrated in this study, machine learning algorithms have the capacity to detect cryptic koalas in a complex and challenging environment without being subject to the same observation biases as traditional detections methods. This can provide some reassurance that these methods provide a robust method for wildlife surveys, and we look forward to additional studies that will contribute to this important topic.

## CONFLICT OF INTEREST

The authors declare no conflicts of interest.

## AUTHOR CONTRIBUTIONS

**Evangeline Corcoran:** Conceptualization (equal); formal analysis (lead); methodology (lead); writing‐original draft (lead). **Simon Denman:** Conceptualization (supporting); data curation (equal); formal analysis (supporting); funding acquisition (supporting); methodology (equal); supervision (equal); writing‐review & editing (equal). **Grant Hamilton:** Conceptualization (lead); formal analysis (supporting); funding acquisition (lead); investigation (lead); methodology (equal); project administration (lead); resources (lead); supervision (lead); writing‐review & editing (lead).

## Data Availability

Datasets used in model development and testing in this study are available from the Zenodo repository https://doi.org/10.5281/zenodo.4620891.
